# Alveolar graft in the cleft lip and palate patient: Review of 104 cases

**DOI:** 10.4317/medoral.19413

**Published:** 2014-06-01

**Authors:** Estela Luque-Martín, María L. Tobella-Camps, Alejandro Rivera-Baró

**Affiliations:** 1 Adjunct Physician in the Department of Orthodontics at Hospital Universitari Sant Joan de Déu, Barcelona; 2 Head of the Department of Orthodontics at Hospital Universitari Sant Joan de Déu, Barcelona

## Abstract

Introduction: Alveolar bone grafting is a vital part of the rehabilitation of cleft patients. The factors that have been most frequently associated with the success of the graft are the age at grafting and the pre-grafting orthodontic treatment. Objectives: 1) Describe the cases of alveolar bone grafts performed at the Maxilofacial Unit of Hospital Sant Joan de Déu, Barcelona (HSJD); and 2) Analyze the success/failure of alveolar grafts and related variables. Material and Methods: Descriptive retrospective study using a sample of 104 patients who underwent a secondary alveolar graft at the Craniofacial Unit of HSJD between 1998 and 2012. The graft was done by the same surgeon in all patients using bone from the iliac crest. Results: 70% of the patients underwent the procedure before the age of 15 (median 14.45 years); 70% of the graft patients underwent pre-graft maxillary expansion. A total of 100 cases were recorded as successful (median age of 14.58 years, 68 underwent pre-graft expansion) and only 4 were recorded as failures (median age of 17.62 years, 3 underwent pre-graft expansion). We did not find statistically significant differences in age at the time of grafting or pre-surgical expansion when comparing the success and failure groups. We found the success rate of the graft to be 96.2%. Conclusions: The number of failures was too small to establish a statistically significant conclusion in our sample regarding the age at grafting and pre-grafting expansion. The use of alveolar bone grafting from the iliac crest has a very high success rate with a very low incidence of complications. Existing controversies regarding secondary bone grafting and the wide range of success rates found in the literature suggest that it is necessary to establish a specific treatment protocol that ensures the success of this procedure.

** Key words:**Alveolar graft, cleft lip and palate, alveolar cleft, alveolar defect.

## Introduction

Seventy-five percent of patients with cleft lip or cleft lip and palate present with an anterior alveolar bone defect that can affect tooth development and contribute to the collapse of alveolar segments. It is therefore necessary to reconstruct the cleft in order to allow for the eruption of adjacent teeth, orthodontic or prosthodontics treatment of the area, and closure of symptomatic oronasal fistulas (1).

Bone grafting currently forms a vital part of the rehabilitation of cleft patients and is included in the treatment protocol at rehabilitation centers ( Table 1).

**Table 1:**
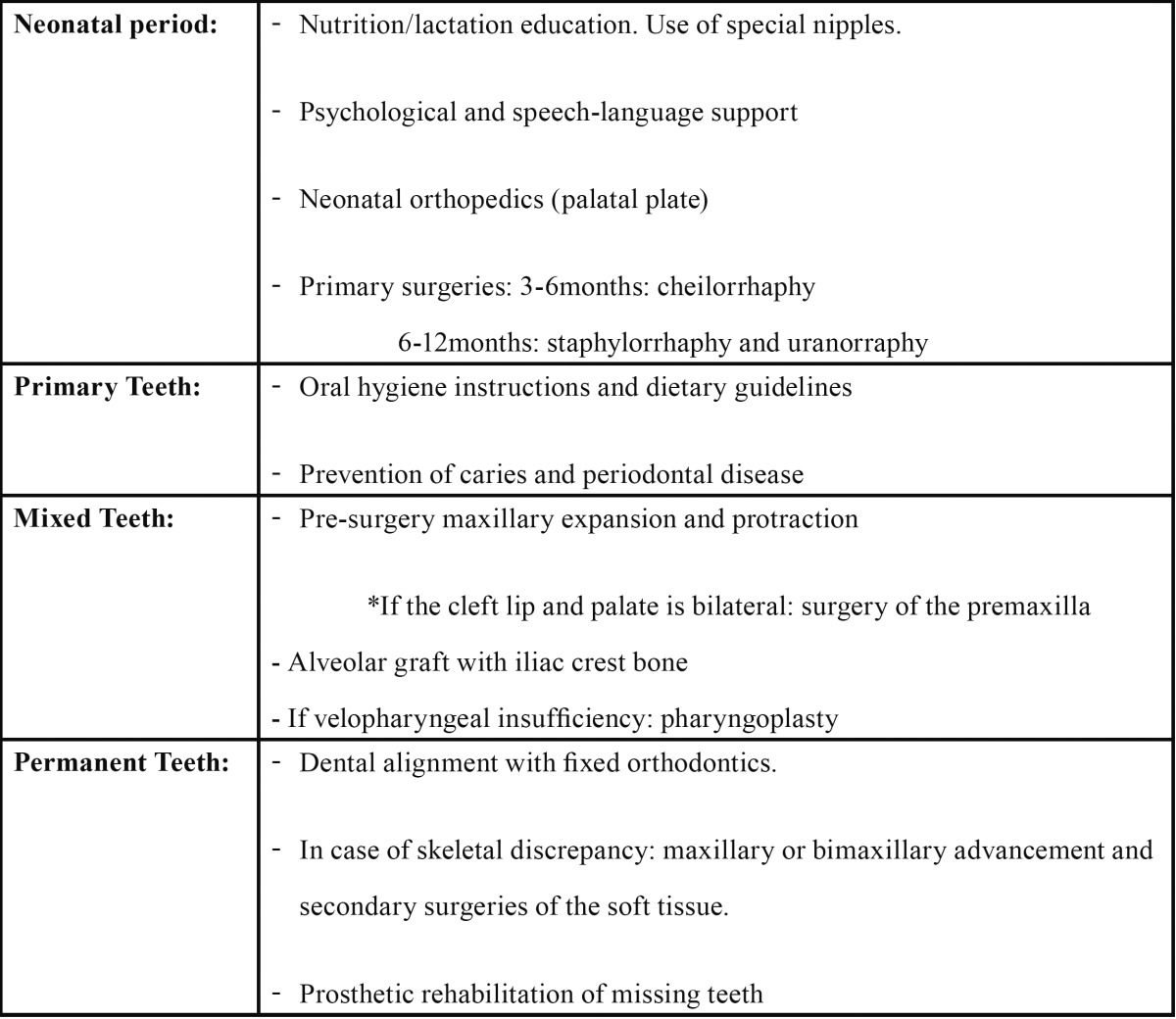
Treatment protocol for the cleft patient at Hospital Universitari Sant Joan de Déu, Barcelona.

The main advantages of performing an alveolar graft are: (1-4)

- To provide osseous support for the teeth near the area of the cleft, thus facilitating eruption of the teeth and improving subsequent orthodontic treatment.

- Uniform formation of the arch and alveolar ridge, simultaneously allowing optimal lip support.

- Closure of the oronasal fistula and improved speech.

- Improvement of facial esthetics: supports nasal structures and lifts the free portions of the nostrils in the area of the cleft, thus improving nasal and labial symmetry.

- Stabilization and partial repositioning of the premaxilla in patients with bilateral clefts.

- To provide adequate bone volume to facilitate subsequent prosthetic rehabilitation with implants.

The issues most commonly causing controversy regarding alveolar bone grafting include: (1-6)

- Timing of the surgical reconstruction: the timing of the procedure is highly variable and currently tends to be performed between 9 and 12 years of age, when the permanent canine is at the height of its eruptive process.

- Orthodontic/orthopedic aspects that affect the area of the cleft, such as maxillary expansion prior to grafting in order to correct anteroposterior discrepancy.

These controversies suggest the need to assess secondary alveolar grafting in order to establish a treatment protocol that ensures the success of this procedure.

The objectives of this study are:

1- To describe the cases of alveolar bone grafts completed at the Maxillofacial Unit at HSJD.

2- To determine the success/failure of alveolar grafts and the related variables (age at grafting and prior expansion).

## Material and Methods

We conducted a descriptive retrospective study of a sample of 104 patients who underwent a secondary alveolar graft at the Craniofacial Unit of Hospital Sant Joan de Déu, Barcelona (HSJD) between 1998 and 2012.

Inclusion criteria included patients who underwent secondary alveolar grafting at the Maxillofacial Unit at HSJD performed by the same surgeon, whose donor site for the graft was the iliac crest, and who had a complete clinical and radiographic record. Patients excluded were those who were not treated at the Maxillofacial Unit at our hospital or were treated by another surgeon, those with donor bone from a site other than the iliac crest, and/or those with incomplete records.

The study looked at clinical histories as well as panoramic radiographs and 2D/3D CT scans of 106 patients, leaving a final sample of 104 due to dismissal of one patient due to missing documentation and another due to usage of a tibial graft.

Information recorded for each patient:

1- Age and sex

2- Type of cleft (left/right lip, unilateral left/right or bilateral lip and palate)

3- Age when alveolar graft was done and its success/failure evaluated clinically and radiographically

4- Orthodontic treatment performed on the patient

5- Ectopic eruption of the maxillary canine and its evolvement

**Figure 1:**
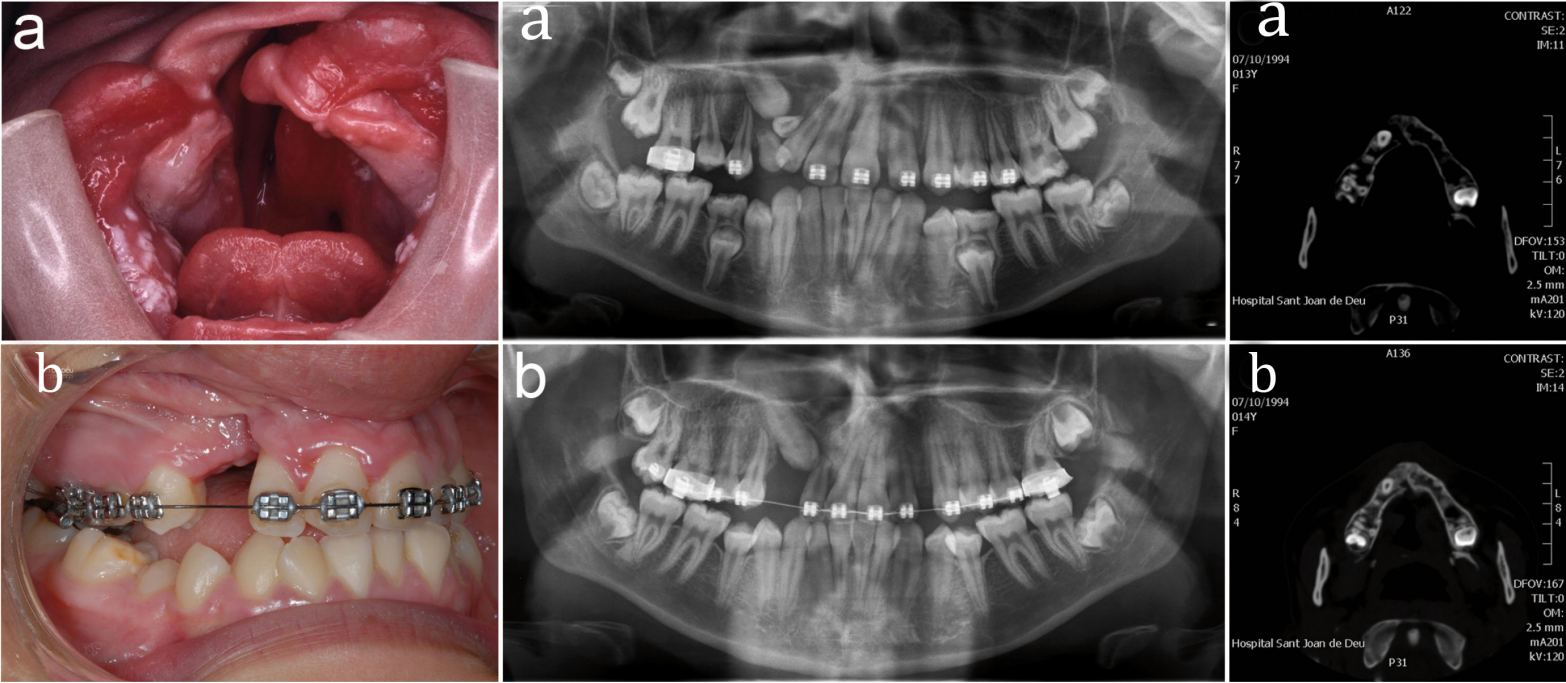
a) clinical and radiographic appearance of the cleft lip and palate b) post-graft clinical and radiographic appearance.

The success of the graft was determined clinically (closure of the fistula, graft inspection and palpation, eruption of the dentition, and stabilization of the adjacent teeth) and radiographically (panoramic radiographs and/or 2D and 3D CT scans at 1 month and 3 months, where we assessed osseous trabeculation and condition of the roots of the adjacent teeth) (Fig. 1).

The surgical protocol used at our center is as follows:

1- Maxillary expansion, if needed (depending on maxillary collapse), to facilitate surgical access. It is completed 6 months prior to grafting. Until the graft has been placed, it is necessary to maintain the roots of the teeth away from the area of the cleft, always surrounded by hard tissue, in order to avoid periodontal damage. Anterior teeth with large rotations that are adjacent to the cleft should not be corrected prior to grafting due to the risk of fenestrations and dehiscences.

2- Extraction of primary teeth and supernumerary teeth adjacent to the cleft at least 6 weeks prior to grafting to avoid possible continuity issues and infection sites that could reduce the possibility of osseointegration.

3- Application of Chlorhexidine gel 3-4 times a day at the cleft site for 2 days prior to surgery.

**Figure 2:**
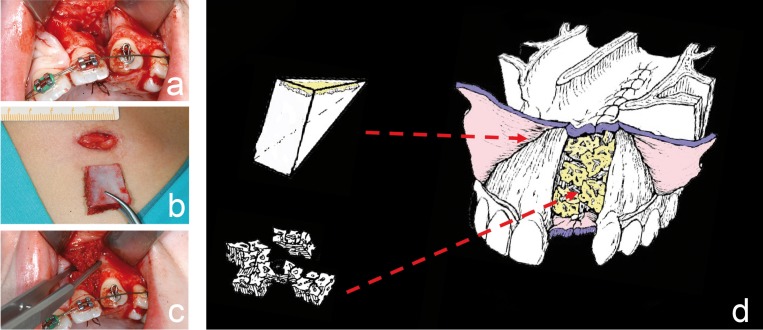
a) Alveolar cleft prior to placement of graft, b) harvesting of a pyramidal bone block from the iliac crest, c) packing of the cancellous bone in cleft area, d) design of the pyramidal corticocancellous graft

4- Alveolar bone grafting (Fig. 2). Our first choice is to use bone from the iliac crest, because it allows harvesting of both cortical and cancellous bone with a low rate of complications. It also makes it possible to harvest the bone and place it in the cleft in a single procedure. The procedure is done under general anesthesia with nasotracheal intubation. Bone is obtained by making a 2 cm long skin incision 1 cm from the anteroposterior iliac spine. It is carried down to the cartilaginous tissue overlying the iliac crest and then an osteotome is used to make 3 cuts: one parallel to the longitudinal axis of the crest and 2 perpendicular ones. In this way, a pyramid-form corticocancellous block is obtained. Subsequently, the repair of the alveolar defect begins with infiltration of articaine in the vestibular and palatal areas of the premaxilla. Two mucoperiosteal advancement flaps are released on the labial side, extending into the cleft. Once there, another incision is made to separate the nasal mucosa from the gingival mucosa. Another similar incision is made on the palate. The nasal mucosa is separated from the lateral wall of the nose and from the oral mucosa in order to lift it towards the floor of the nose (the nasal floor is sutured with 4-0 polyglactin suture). The pyramidal block of bone previously harvested is grafted into the cleft in a manner in which the cortical surface that will form the nasal floor comes into contact with the nasal mucosa. The other cortical surface will form the vestibular cortical plate (very important for future implant placement). The remaining spaces in the cleft are formed using cancellous bone. Finally, the gingival tissue is closed with 4-0 polyglactin suture with reapproximation of the papilla.

5- Rigorous hygiene, application of chlorhexidine gel on the surgical wound for 15 days. Valsalva maneuvers should not be performed.

6- Postoperative evaluation after 15 days, 1 month, and 3 months (with checkup CT and panoramic radiograph).

7- Orthodontic treatment is resumed approximately 6 weeks later. Treatment with fixed appliances is initiated to align the arches, and the expansion achieved prior to the graft is retained between 6 and 12 months. In most cases, the expander is substituted with a palatal bar as a retainer of the expansion.

## Results

We studied a total of 104 patients, of which 73 (70%) were males and 31 were females (30%).

The distribution of the clefts was as follows:

-Bilateral cleft lip and palate: 22 (22%)

-Cleft lip and palate: 63 (60%) (43 left, 20 right)

-Cleft lip: 19 (18%) (13 left, 6 right)

Fifty percent of the sample (52 patients) initiated their treatment at our center from birth. Of those, 36 patients (69%) used a palatal plate. Cleft lip patients did not use a palatal plate, and of those with cleft lip and palate, only 4 did not use a plate.

**Figure 3:**
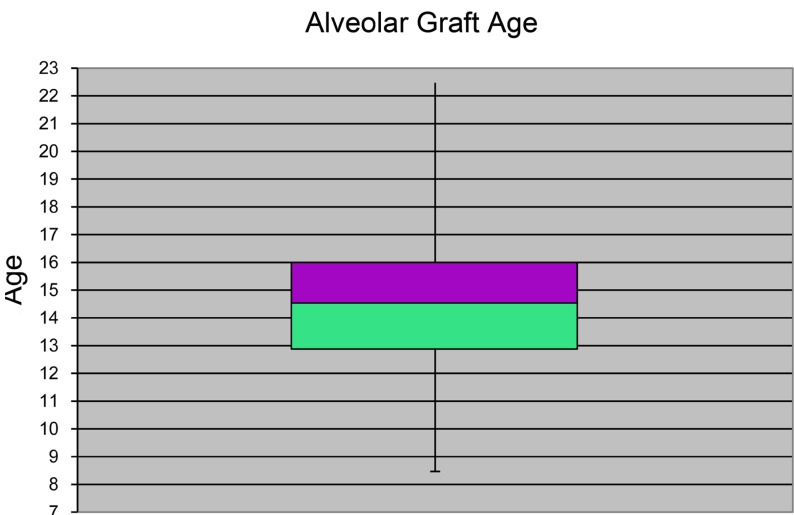
Age at which alveolar grafting is done.

The median age at which the graft was done was 14.45 years, with a range of 8.5 years to 22 years. Due to the large range in ages within the sample, we separated the age ranges as follows ( Table 2) (Fig. 3):

**Table 2:**
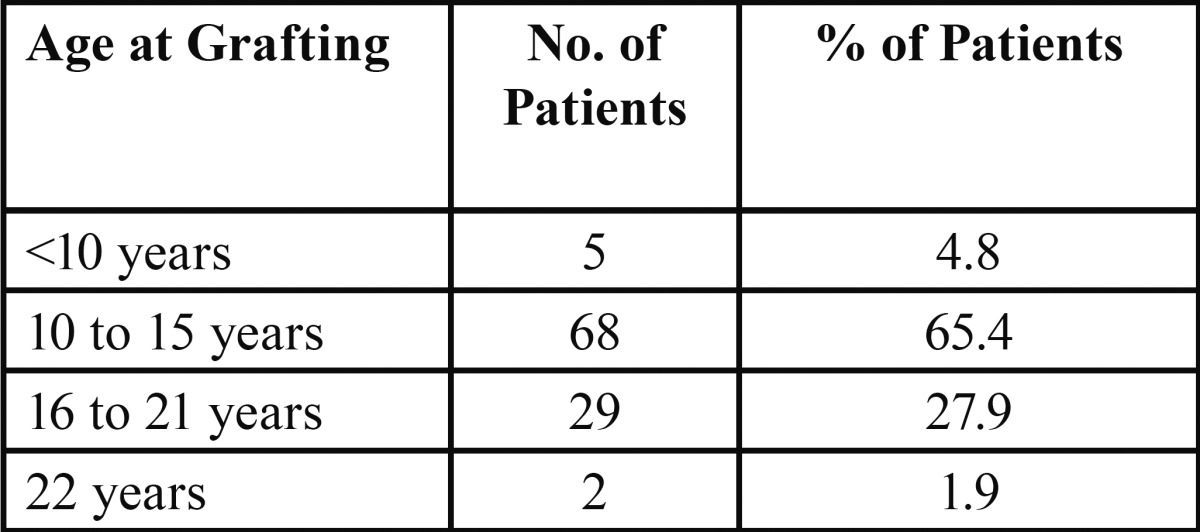
Age at which alveolar grafting is done: distribution by age ranges.

Of the 104 patients, 71 underwent pre-graft expansion (68.2%). The following table ( Table 3) presents the different options for expansion and the frequency with which each one was used:

**Table 3:**
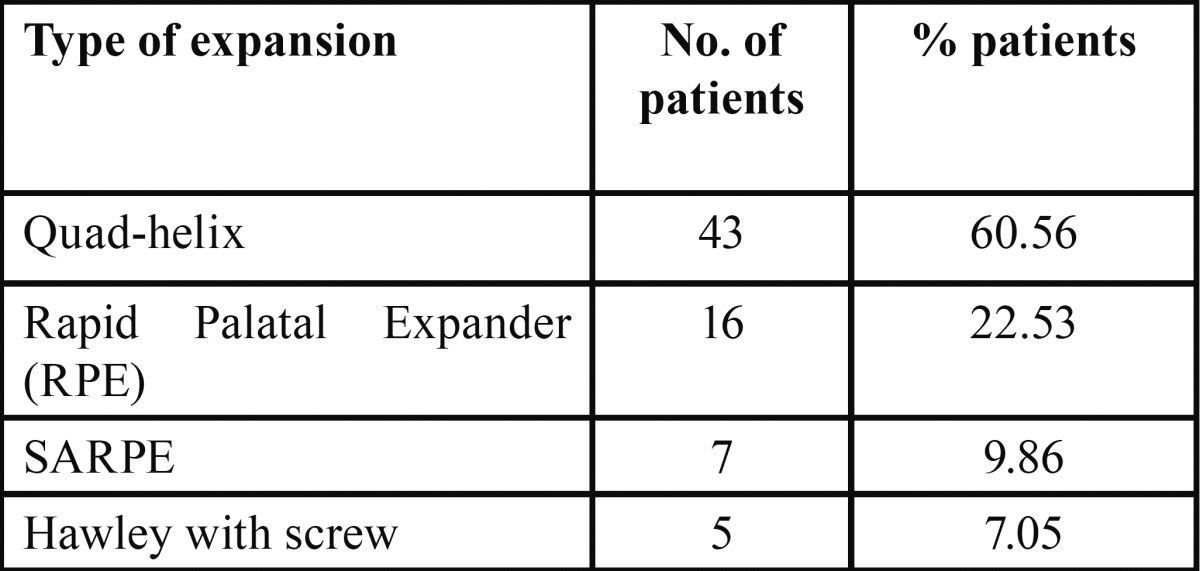
Options for expansion and their frequency of use.

Another orthodontic treatment completed prior to grafting was the use of a facemask to improve anterior crossbite of skeletal origin. Fourteen patients wore a facemask during the initial phase of orthodontics; in 7 of those patients, it was combined with a quad-helix expander, in 3 with an RPE, in 3 with SARPE, and 1 patient wore the mask with no other type of expansion.

Of the 104 patients, a total of 34 ectopic canines were recorded (in 32 patients), of which 25 were guided into occlusion, 8 erupted spontaneously after the graft, and only 1 had to be extracted.

Of the total in our sample (104), the graft was considered a failure in only 4 patients (3.8%), which thus provides us with a success rate of 96.2%. The median age at grafting for the success group (n=100) was 14.58 years and for the failure group (n=4) was 17.62 years. The results obtained by the Mann-Whitney U test (U=68) do not allow us to confirm whether there are statistically significant differences with a significance level of *p*≤0.05.

As far as the pre-graft expansion, if we compare the two groups, in the success group, 68 patients underwent expansion prior to the graft, and in the failure group, 3 patients. Again, we did not find significant differences between the two groups by the use of the statistical chi-squared test and the correction by the Fisher test for frequencies smaller than 5 (due to the failure group being n=4) with a significance level of *p*≤0.05.

With regards to complications associated with the graft, 1 patient presented with perialar infection that resolved with antibiotic treatment. As far as the donor site, 1 case required scar retouching.

## Discussion

Since its description in the seventies, it has been acknowledged that alveolar grafting is an essential part of treatment in patients with cleft lip or cleft lip and palate. During that decade, primary alveolar grafting (before 3 years of age) was being done frequently, until Koberg and Ross described its adverse effects, such as restriction of maxillary growth or deficient alveolar morphology with unerupted teeth or teeth with no support (1,3).  Table 4 summarizes the main advantages and disadvantages of primary and secondary grafts.

**Table 4:**
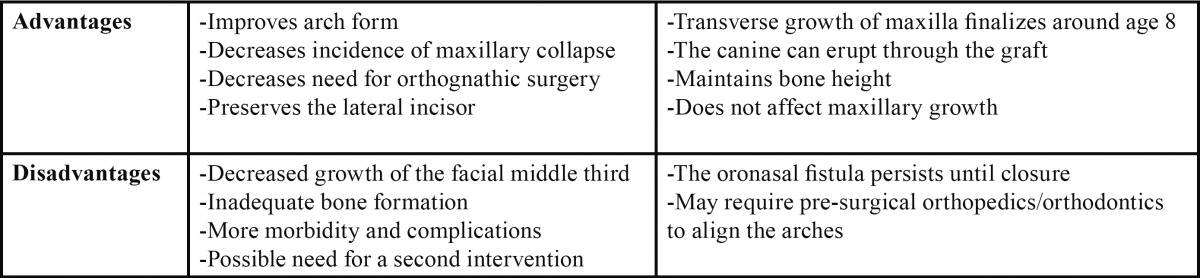
Advantages and disadvantages of primary and secondary grafts (3).

Nevertheless, any surgical procedure can cause alterations in bone growth and these alterations are more severe the earlier the procedure is performed. For this reason, Kujipers-Jagtman and Long concluded that a detailed study of every case is needed to determine the risks and benefits of the procedures (2).

Many studies have shown that the success rate of grafts is lowered if the procedure is done after the eruption of the canine on the side of the cleft. Once the tooth has erupted, improvement in the periodontal support of the tooth cannot be expected with the placement of the graft. For this reason, it is recommended that the graft be placed before the eruption of the permanent canines (2).

Nowadays, the graft is typically placed between 8 and 12 years of age, when the permanent canine is located high in the alveolar process (with 1/3 or 1/2 completed root formation) at the height of the eruptive process (7).

In our study, 70% of the completed grafts were done before age 15. The rest of the grafts were done after age 16 and were related to transfers from other centers (which meant that the patient entered into our treatment protocol at a more advanced age) or with previous preparation for implant placement in the anterior region. There is a difference of 3 years between the median age at grafting of the success group (14.58) and the failure group (17.62); however, we could not find statistically significant differences due to the low number of failures.

As far as orthodontic treatment, maxillary expansion tends to be included in the majority of treatment protocols for cleft lip and palate patients. At our hospital, it is done in a systematic manner with patients who present with maxillary collapse (68.2%). In the success group, it was performed in 68 of the 100 patients, and in the failure group, it was performed in 3 of the 4 patients.

The most commonly used appliance is the quad-helix, as it is the simplest to adapt to the collapsed arches of cleft patients, where in many cases there is not enough room to place an expansion screw for an RPE appliance. In cleft arches (with already separated palatal processes), and at the age at which the expansion is done, it is possible to obtain results similar to those of an RPE with this type of appliance. The RPE is indicated when there is a bilateral posterior crossbite and when it is physically possible to place it. The most severe compression cases (where expansion greater than 7 mm is needed) are treated surgically with SARPE. The mildest cases (unilateral edge to edge occlusion) can be treated with a Hawley with an expansion screw.

Maxillary expansion prior to grafting favors anterior nasal projection, improves growth potential, increases the percentage of spontaneously erupted canines, and facilitates surgical access (5,6,8,9,10).

Nevertheless, there are few studies in the literature that link orthodontic treatment to alveolar graft success. In one study done in the United Kingdom by various member centers of CLEFTSiS between the years 2000 and 2004, it was observed that the success of the graft was significantly higher in patients that underwent prior palatal expansion than in those who did not. This is because the reopening of the alveolar cleft means that the floor of the nose can be corrected simultaneously and a larger volume of bone can be grafted, preventing perinasal bone loss (11).

One study conducted at the Shariati Hospital in Tehran between 2005 and 2008 concluded that the only factor significantly associated with the success of alveolar grafts was orthodontic treatment. Ninety-one percent of patients who underwent maxillary expansion prior to the surgery were associated with graft success (9).

If active expansion is done after the graft, an opening of the palatal suture is possible, but results are unpredictable and there is an increased probability for appearance of fistulas (6,9). Once the pre-grafting expansion is completed, it is important to retain it with a palatal bar or the placement of brackets to avoid possible recurrence or maxillary collapse (6). Seeing as post-graft orthodontics is also linked to the success of alveolar grafts, Kindelan and Robert Harry (12) found a success rate of 63.2% in patients with alveolar cleft that continued orthodontic treatment. Those who did not continue with the orthodontics had a success rate of 40%.

In our study, we recorded only 4 failures of total graft patients. In one case, bone sequestration was associated with sinus inflammation, and in another, it was associated with bad oral hygiene and tobacco use. In these patients, the bone fragment was removed and regrafted starting a year after the first intervention. We thus found a success rate of 96.2%, similar numbers to those found in the literature.

Tan *et al.* analyzed a series of 100 iliac graft cases between 1982 and 1987. The age at the intervention ranged between 8 and 20 years. They found a success rate of 98% and attributed the failures to postsurgical infections related to patients’ bad oral hygiene (13).

In one study published in 2012, Toscano *et al.* report a success rate of 91.84% in a sample of 49 patients with an age range of 8 to 14 years. They did not associate the success rate with the type/width of the cleft or to sex, age, or agenesis of lateral incisors. The only factors involved were dental age at grafting and orthodontic treatment completed before and after the graft (6).

Long *et al.* also obtained a success rate of 91% in their study conducted on a sample of 43 patients that underwent an alveolar graft at a median age of 11.1 years (8.6 to 16.9) (4).

Other success rates recorded in the literature are: 87% in a study done at The Hospital for Sick Children in London between 1982 and 1989 with a sample of 115 patients (14); 73% in a study by Kindelan *et al.* conducted between 1992 and 1995 with a sample of 38 patients (15); and 72% in a study of 50 patients by Da Silva Filho *et al.* (10).

The wide range of success rates found in the literature suggests that it is necessary to establish a specific treatment protocol to ensure the success of this procedure. It is difficult to establish a comparison between the various studies published, due to the great variety of treatment protocols applied, the different criteria used to evaluate the grafts (clinical, radiographic, or both), and the sizes of the study samples.

## Conclusions

-The number of failures is too small to be able to establish any sort of statistically significant conclusion regarding the age at grafting and pre-grafting expansion in our sample.

-The use of alveolar bone graft from the iliac crest achieves a very high rate of success and has a very low incidence of complications.

-Existing controversies regarding secondary bone grafting and the wide range of success rates found in the literature suggest that it is necessary to establish a specific treatment protocol to ensure the success of this procedure.
